# The antitumor potential of *Polygonatum* spp.: a narrative review of traditional uses, bioactive metabolites, and multi-targeted mechanisms

**DOI:** 10.3389/fphar.2025.1662839

**Published:** 2025-11-10

**Authors:** Mengqin Zhu, Guang Chen, Jinyu Li, Chang Yi, Yanfeng Yuan, Wenlong Liu, Xili Zhang

**Affiliations:** 1 School of Pharmacy, Hunan University of Chinese Medicine, Changsha, China; 2 Hunan Provincial Key Laboratory of Drugability and Preparation Modification of TCM, Changsha, China; 3 Hunan Hengyang Hospital of Traditional Chinese Medicine, Hengyang, Hunan, China

**Keywords:** Polygonatum spp., traditional uses, antitumor activity, bioactive metabolites, multi-targeted mechanisms, pharmacological properties

## Abstract

*Polygonatum* spp., encompassing various species within the genus, is a traditional Chinese botanical drug. It is known for its pharmacological effects, including qi tonification, yin nourishment, spleen invigoration, lung moistening, and kidney tonification. *Polygonatum* contains abundant bioactive metabolites, such as polysaccharides, steroidal saponins, flavonoids, volatile metabolites, and alkaloids. Recent research primarily revolves around its anti-inflammatory, anti-ageing, and glycaemic regulatory properties, while its antitumor potential remains comparatively underexplored. Malignant tumors represent a considerable global public health obstacle and are now a leading contributor to the global disease burden. The identification of effective antitumor agents and therapeutic strategies is urgent. Bioactive metabolites in *Polygonatum* have shown strong cytotoxic and pro-apoptotic impacts *in vitro* and *in vivo*. However, current research mostly focuses on isolated metabolites, lacking comprehensive narrative analysis. This review endeavors to narratively summarize recent advances on the antitumor activity and underlying mechanisms of *Polygonatum*, critically evaluate existing research gaps, and proposes future directions to facilitate the development of *Polygonatum* as a potential novel anticancer agent.

## Graphical Abstract

**Figure:**
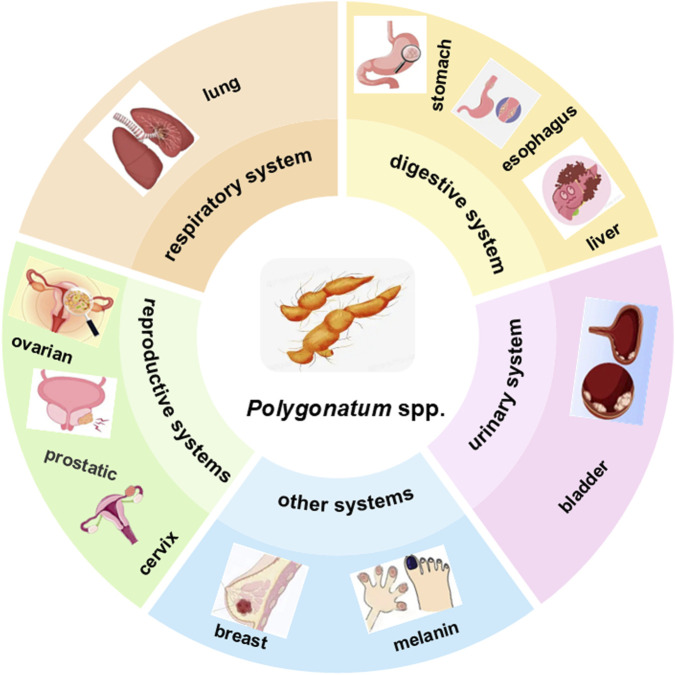

## 1 Introduction

Cancer, as a major class of diseases that seriously threaten human health, has long been a research priority in the global medical field. In recent years, due to environmental changes, lifestyle shifts and the accelerated ageing of the population, the incidence and mortality rates of cancer have shown a sustained upward trend (Łaniewski et al., 2020). According to the World Health Organization, there were as many as 19.3 million new instances of cancer globally in 2020, resulting in nearly 10 million deaths (Chhikara and Parang, 2023). In China alone, there were 4.57 million new cancer diagnoses and 3 million cancer-related deaths in 2020, with morbidity and mortality rates among the highest globally. Currently, clinical therapies for cancer primarily include surgery, chemotherapy, radiotherapy, targeted therapy, and immunotherapy. While these approaches have improved survival rates and quality of life for cancer sufferers to some extent, challenges remain, such as limited applicability to certain populations, drug resistance, and high toxicity and significant side effects (Liu et al., 2024a). These challenges are closely related to the multifactorial nature of tumorigenesis, which involves genetic mutations, epigenetic changes, dysregulated signaling pathways, and tumor–immune interactions. Tumor development generally proceeds through initiation, promotion, progression, and metastasis (Testa et al., 2018; Zhang et al., 2024), with immune cells such as regulatory T cells, macrophages, and NK cells playing important roles (Lei et al., 2020). This complexity highlights the need for therapies that can target multiple pathways simultaneously, consistent with the principles of traditional Chinese medicine (TCM).

TCM has a long-standing history and extensive practical experience in cancer treatment, owing to its multi-metabolites, multi-target, and holistic regulatory properties (Chan, 1995). *Polygonatum* spp., a traditional medicinal and edible substance, has been used in China for over 2,000 years. As recorded in the *Compendium of Materia Medica*, it “nourishes the middle and benefits the qi, removes wind-dampness, pacifies the five viscera, lightens the body, and prolongs life when taken for a long time”. This botanical drug is characterized by its sweet taste and neutral nature, and it is attributed to the spleen, lung, and kidney meridians. It is renowned for its capacity to replenish qi, nourish yin, enhance spleen function, moisten the lungs, and support kidney health. Modern research has identified numerous chemical metabolites in *Polygonatum* spp., such as polysaccharides, steroidal saponins, flavonoids, and amino acids, which exhibit antioxidant, anti-inflammatory, antibacterial, hypoglycemic, hypolipidemic, and immunomodulatory effects (Zhao et al., 2018). Recent studies also suggest that *Polygonatum* spp. May inhibit tumor cell proliferation, induce apoptosis, and enhance immunity (Liu et al., 2024b). However, most investigations have focused on single metabolite, and its mechanisms remain insufficiently clarified. Therefore, this paper aims to summarize the main antitumor metabolites of *Polygonatum* spp. and their mechanisms of action, analyze current research progress, and identify existing challenges, thereby providing a foundation for its further development and application as a natural anticancer agent.

## 2 Traditional uses of *Polygonatum* spp.

*Polygonatum* spp(Huangjing) has a documented history of over 2,000 years in China, and is classified as a superior botanical drug for tonifying qi, nourishing yin, and strengthening the spleen, lungs, and kidneys. These properties were first described in ancient texts such as the *Shennong’s Classic of Materia Medica* and later elaborated in the *Compendium of Materia Medica* (Xu et al., 2021). Subsequent materia medica also consistently emphasized its use in “deficiency syndromes,” a concept encompassing fatigue, weight loss, night sweats, and weakness of the immune system—symptoms that closely resemble those frequently observed in cancer patients (Su et al., 2023). *Polygonatum* preparations were prescribed for the management of sores, ulcers, and protracted wounds, conditions that may reflect chronic inflammation and tumor-related complications. Such long-recognized applications suggest potential anti-inflammatory, immunomodulatory, and restorative activities (Wang et al., 2022).

Modern pharmacological investigations have increasingly corroborated these traditional claims. Recent studies have identified polysaccharides, flavonoids, and saponins as the main bioactive metabolites, which exert notable antitumor effects via diverse mechanisms, including enhancement of immune responses, induction of cancer cell apoptosis, and suppression of tumor proliferation. Thus, the convergence of traditional knowledge with modern pharmacological evidence highlights *Polygonatum* spp. as a promising candidate for integrative cancer therapy.

## 3 Antitumor active metabolites of *Polygonatum* spp.

**FIGURE 1:**
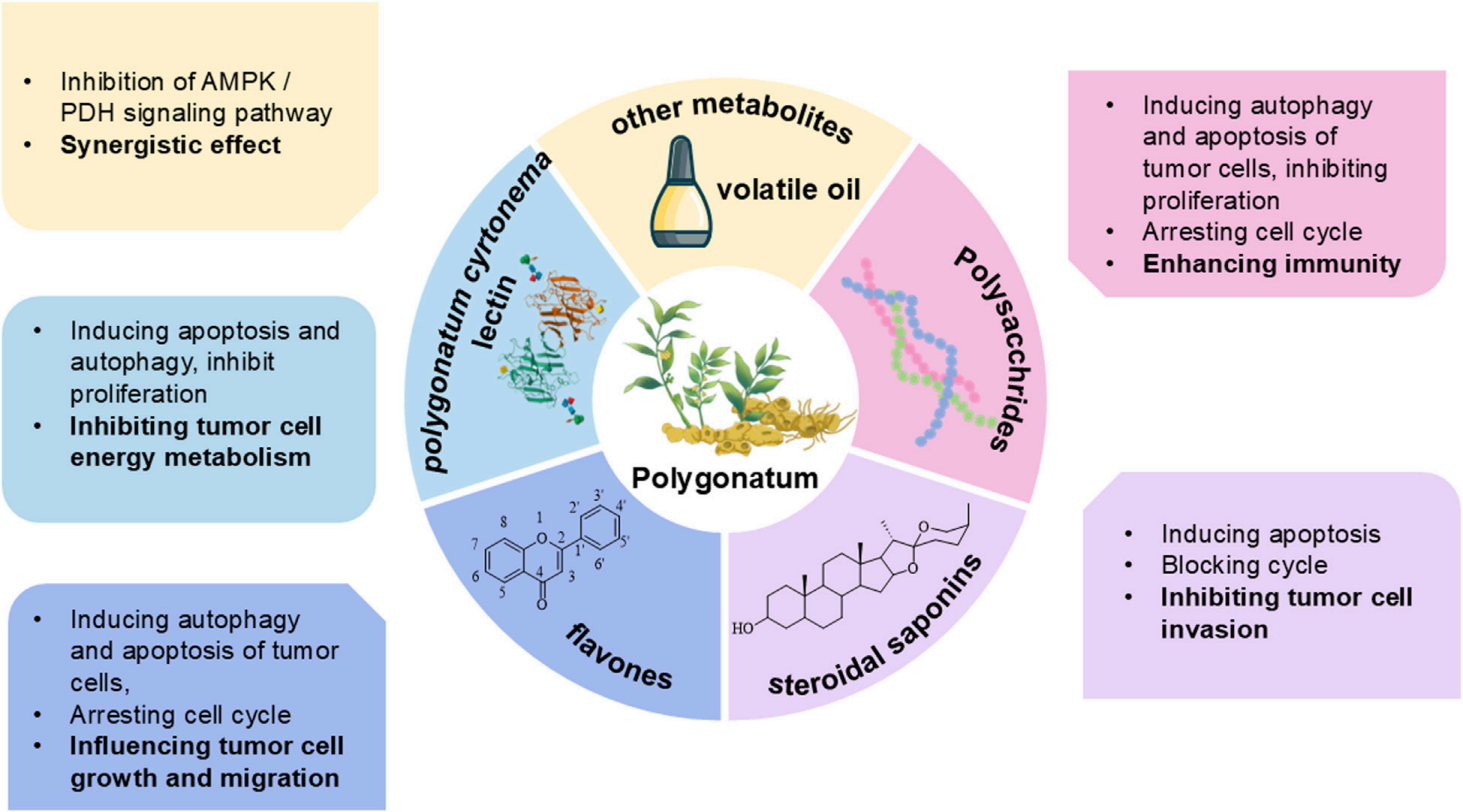
The antitumor metabolites of *Polygonatum* spp.

As a traditional Chinese botanical drug, *Polygonatum* spp. (Huangjing) is characterized by complex metabolites, with its principal active metabolites including polysaccharides, saponins, flavonoids, anthraquinones, alkaloids, amino acids, and volatile oils (Ren et al., 2020). Modern research indicates that the metabolites primarily responsible for its antitumor activity include polysaccharides, steroidal saponins, flavonoids, and *Polygonatum cyrtonema* lectin. The active antitumor metabolites of *Polygonatum* spp. and the different molecular mechanisms through which they exert their effects in cancer treatment are illustrated in Figure 1.

### 3.1 *Polygonatum* polysaccharide

*Polygonatum* polysaccharide (PSP) is the most abundant and pharmacologically active metabolites in *Polygonatum* spp., serving as both a quality marker and the primary active metabolite. It demonstrates abundant biological activities, including antioxidant, anti-aging, glucose and lipid metabolism regulation, immunomodulation and anti-tumor effects (Hu et al., 2023). Recent research has indicated that PSP can inhibit the growth of extensive types of tumors, like liver, lung, gastric, breast, and cervical cancers. Its mechanisms of action involve inducing tumor cell apoptosis, inhibiting tumor cell proliferation, blocking the cell cycle, and activating the immune response (Lai et al., 2024). In cervical cancer HeLa cells, PSP fractions suppressed proliferation and induced apoptosis in a concentration-dependent manner, with concentrated alkali-soluble solids (CASS) displaying the strongest effect by regulating apoptosis-related gene expression (Li, 2019). PSP also prolonged the survival of S180 ascitic tumor-bearing mice, and inhibited H22 solid tumors (Zhang et al., 2007). Moreover, PSP enhanced immune function by activating the TLR4–MAPK/NF-κB pathway, thereby promoting cytokine secretion and facilitating tumor cell clearance (Long, 2018).

### 3.2 Steroidal saponins

Steroidal saponins are synthesized through the combination of steroidal saponin aglycones with sugars, which mainly include D-glucose, D-galactose, D-xylose, L-rhamnose, and L-arabinose. Recent research has shown that steroidal saponins are the principal bioactive metabolite of *Polygonatum* spp., exhibiting a wide range of pharmacological properties, including anti-inflammatory, antibacterial, and lipid-lowering effects, as well as notable antitumor activity (Xu et al., 2023). Recent research has demonstrated that metabolites such as flavnoide B, polygonum saponin, and diosgenin in *Polygonatum* spp. Exhibit potent therapeutic efficacy against various cancers, including lung cancer, hepatocellular carcinoma, breast cancer, melanoma, and cervical cancer. These metabolites primarily restrain tumor cell proliferation, trigger apoptosis, and curb tumor invasion (Zhou et al., 2019a; Sharma et al., 2021; Xie and Chen, 2021). Diosgenin demonstrated inhibitory effects on human epidermoid carcinoma cells, showing an activity comparable to adriamycin, as determined by the thiazolyl blue method (Zhang et al., 2007). Purified steroidal saponins have been reported to suppress A2780 ovarian cancer cell proliferation by promoting apoptosis, disrupting the cell cycle, modulating intracellular reactive oxygen species (ROS) levels, and regulating pro- and anti-apoptotic proteins, achieving an inhibition rate of 66.76% (Xia et al., 2025). Spirosteroidal saponins from *Polygonatum* spp., including *Polygonatum sibiricum* Redouté [*Asparagaceae*; *Polygonati rhizoma*] saponin B and dioscin, exhibited notable activity against various tumor cell lines, such as HL-60, 7901, A549, KB, and HeLa. Among these metabolites, diosgenin demonstrated the strongest activity, significantly inhibiting the growth of murine sarcoma S180 and murine hepatocellular carcinoma HAC (Yang, 1999).

### 3.3 Flavonoids

Flavonoids are a category of natural organic metabolites characterized by a C6-C3-C6 structure, with a basic nucleus of 2-phenylchromone. Based on the degree of oxidation of the central three-carbon chain, the attachment of the B ring (at the 2 - or 3 - position) and whether the three-carbon chain is ring-forming and other characteristics, flavonoids can be classified into flavonoids, flavonols, dihydroflavonoids, isoflavonoids, chalcones, and other types of metabolites (Taldaev et al., 2022). Flavonoid metabolites display a broad spectrum of biological activities, including antioxidant, anti-aging, anti-inflammatory, immunomodulation, and cardiovascular protection. Among these metabolites, high-isoflavonoids, chalcones, dihydroflavonoids, and flavonoid glycosides have demonstrated significant anticancer activities (Tao et al., 2018). These metabolites inhibit tumor development by interfering with the cell cycle, inducing apoptosis and autophagy, and modulating related signaling pathways, thereby impacting the development and metastasis of tumor cells (Ye et al., 2025). Two high-isoflavonoids were isolated from *Polygonatum odoratum* (Mill.) Druce [*Asparagaceae*; *Polygonati rhizoma*] and were found to induce Bcl-2 phosphorylation, trigger apoptosis, and arrest the G2/M phase of the cell cycle in breast tumor cells (Rafi and Vastano, 2007). Chemical metabolites from the rhizomes of *Polygonatum kingianum* Collett & Hemsl. [*Asparagaceae*; *Polygonati rhizoma*] were isolated and characterized, revealing four novel high-isoflavones that effectively suppressed the growth of HepG2 cells and non-small cell carcinoma cells associated with hepatocellular carcinoma, and their findings revealed that four novel high-isoflavones effectively suppressed the growth of HepG2 cells and non-small cell lung cancer cells associated with hepatocellular carcinoma. Notably, the metabolite (3R)-5,7-dihydroxy-8-methyl-3-(2′-hydroxy-4′-methoxybenzyl)-chroman-4-one demonstrated significant inhibitory activity against mouse tumor cells. Additionally, the IC_50_ value of this metabolite for mouse macrophages was determined to be 17.99 ± 1.45 μmol/L (Xuan et al., 2025).

### 3.4 *Polygonatum cyrtonema* lectin

*Polygonatum cyrtonema* lectin (PCL) is a mannose/sialic acid-binding lectin isolated from the rhizome of *Polygonatum cyrtonema* Hua [*Asparagaceae*; *Polygonati rhizoma*], which has been shown to possess multiple anti-tumor properties, such as inducing apoptosis and autophagy, and inhibiting migration (Liu et al., 2016). Studies indicate that PCL can induce tumor cell apoptosis through activation of the caspase and mitochondrial ROS-p38-p53 signaling pathways. Meanwhile, it inhibits the Ras-Raf and PI3K-AKT pathways to enhance autophagy and promote cancer cell death (Wang et al., 2011). Lectins isolated from *Polygonatum multiflorum* (L.) All. [*Asparagaceae*; *Polygonati rhizoma*] were reported to significantly inhibit the malignant progression of A375 melanoma cells by simultaneously modulating autophagy and apoptosis pathways (Liu et al., 2009b). Mechanistic analysis revealed that PCL family member PCL-2 promoted apoptosis by enhancing ROS production, upregulating the expression of pro-apoptotic genes (Bax, Caspase-3, and Caspase-9) at both mRNA and protein levels, and downregulating the expression of the anti-apoptotic gene Bcl-2, thereby suppressing tumor cell proliferation (Sun et al., 2022).

### 3.5 Other metabolites

To further complement the antitumor profile of *Polygonatum* spp., beyond the primary bioactive metabolites discussed above, various secondary metabolites such as phenolic acids, alkaloids, volatile oils, and lignans have also been shown to exert immunomodulatory and antitumor effects through multi-target regulation of key signaling pathways (Song et al., 2024; Wan et al., 2024). Volatile oils extracted from *Polygonatum cyrtonema* Hua demonstrated significant cytotoxic activity against NCI-H460 human lung cancer cells *in vitro*, achieving an inhibition rate of 98.08% at a concentration of 100 μg/mL (Yu et al., 2008). The anticancer potential of various solvent fractions of *Polygonatum verticillatum* (L.) All. [*Asparagaceae*; *Polygonati rhizoma*] was assessed, indicating that dichloromethane, chloroform, and aqueous extracts exhibited dose-dependent cytotoxicity against cancer cells at concentrations ranging from 25 to 400 μg/mL, with the chloroform extract showing the most potent cytotoxic effect (Singh and Patra, 2018). Further studies have highlighted the synergistic interactions among multiple phytochemicals within *Polygonatum sibiricum* Redouté extracts. The identified metabolites were shown to inhibit the AMPK/PDH signaling pathway, suppress mitochondrial oxidative phosphorylation in M2 macrophages, impede M2 polarization, and facilitate the phenotypic switch to the M1 macrophage subtype. These actions collectively inhibit tumor cell migration and enhance immune surveillance (Hou, 2023). Additionally, UPLC-Q-Exactive-MS, network pharmacology, and molecular docking were applied to analyze the chemical metabolite of *Polygonatum sibiricum* Redouté leaves, identifying 56 metabolites, three core bioactives, 11 major targets, and 30 KEGG pathways related to antitumor effects. This integrative study elucidated the multi-metabolites, multi-target, and multi-pathway mechanisms underlying the antitumor activity of the extracts (Chen et al., 2023).

## 4 Mechanisms of antitumor effects of active metabolites in *Polygonatum* spp.

**FIGURE 2:**
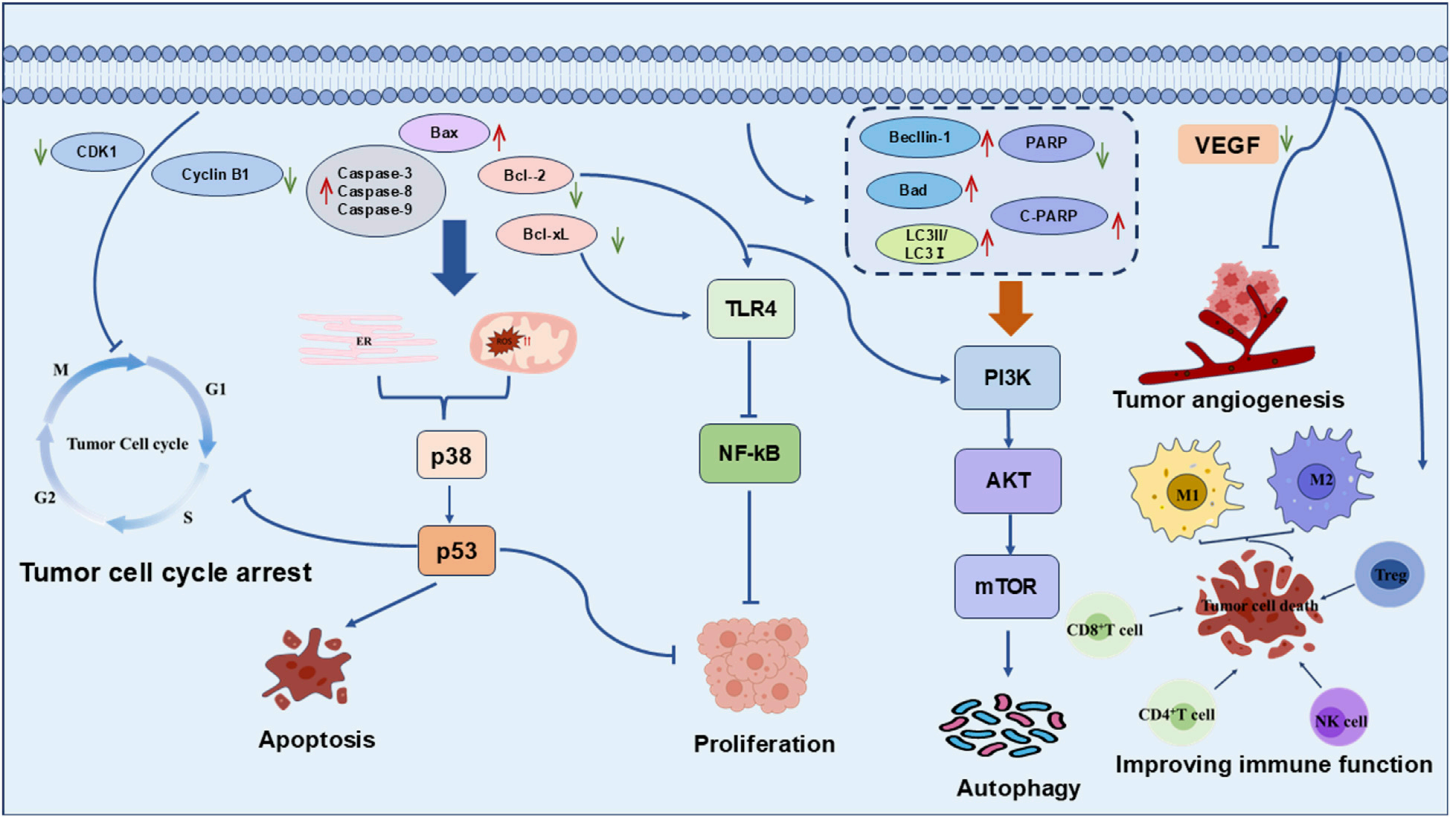
Antitumor mechanism of the active metabolites of *Polygonatum* spp. The antitumor mechanisms of *Polygonatum* spp. Mainly involve cell cycle arrest, apoptosis induction, proliferation inhibition, angiogenesis suppression, autophagy promotion, and immune modulation.

Recent studies indicate that the anti-tumor mechanisms of the bioactive metabolites of *Polygonatum* spp. are multifaceted and synergistic, involving cell cycle arrest, stimulation of tumor cell apoptosis, promotion of autophagy, modulation of the tumor microenvironment, and immune system regulation. These pathways collectively facilitate the suppression of tumor development and progression, as shown in Figure 2. The specific metabolites and their corresponding mechanisms are summarized in Table 1.

**TABLE 1:** Antitumor active metabolites and mechanism of action of *Polygonatum* spp.

Metabolites	Study type	Cancer model	IC_50_/Dose	Pathway targeted	Reference
PSP	*In vitro*	Cervical cancer Hela cells	The IC_50_ of HBSS, CHSS, DASS and CASS were 532.528, 574.028, 655.225, 456,801 μg/mL	Blocking in G2/M phase	Li (2019)
	*In vivo*	S180 ascites tumor mice	100, 200, 400 mg/kg/d for 10 days	Enhancing the immune function	Zhang et al. (2007)
	*In vitro* an *in vivo*	RAW264.7 cells; Lewis lung carcinoma tumor-bearing mice	200 μg/mL, 24 h; 200 mg/kg/d	Activating TLR4-MAPK/NF-κB signaling pathway enhancing immunoregulation	Long (2018)
	*In vivo*	H22 liver cancer mouse model	100, 200, 400 mg/kg/d, for 10 days	Blocking in G0/G1 phase, activating Caspase family to induce apoptosis in tumor cells	Duan et al. (2014)
	*In vitro*	HepG2 liver cancer cells	100, 200, 400 μg/mL L for 72 h	Inducting apoptosis via the mitochondrial pathway	Li et al. (2022)
	*In vitro*	ESCC cells	50, 100, 200, 400 μg/mL	Inhibiting NF-κB signaling	Zhou et al. (2019b)
	*In vitro*	CAFs cells	2, 10, 50, 250, 1,250 μg/mL for 48 h	Stimulating LC 3 II and Beclin-1 expression, promoting autophagy in CAFs	Han et al. (2016)
	*In vivo*	Tumor-bearing mouse model of liver cancer	200, 700 mg/kg/d for 30 days	Inhibiting of VEFG production	Zhang (2025)
	*In vivo*	4T1 TNBC mice model	300 mg/kg/d for 15 days	Enhancing the immune function	Xie et al. (2021)
	*In vivo*	MFC gastric cancer-bearing mice	100, 400 mg/kg/d for 30 days	Inhibiting the TLR4/NF-κB signaling pathway	Lv and Duan (2020)
	*In vivo*	LLC lung cancer-bearing mice	200 mg/kg/d for 20 days	Activating the TLR4-MAPK/NF-κB signaling pathway	Long et al. (2018)
	*In vivo*	Prostate cancer mouse model	100, 200, 400 mg/kg/d for 30 days	Inhibition of PI3K/AKT, and NF-κB signaling pathway	Yang et al. (2025)
Steroidal saponin	*In vitro*	A2780 ovarian cancer cells	150, 300, 600 μg/mL for 24 h	Increasing ROS levels, disrupting the cell cycle and promoting apoptosis	Xia et al. (2025)
Dioscin	*In vitro*	MCF-7 breast cancer cells	1, 1.25, 1.5 μg/mL for 48 h	Up-regulating the expression levels of P53, Caspase-3, Caspase-9 and Bax, and down-regulating the expression level of Bcl-2 protein	Luo (2024)
	*In vivo*	Endometrial cancer mice model	24 mg/kg	Regulating PI3K/AKT/mTOR signaling pathway and p53 pathway	Li et al. (2024)
	*In vitro*	Hepatocellular carcinoma HepG2 cells Cervical cancer Hela cells	IC_50_ = 8.34 μmol/L IC_50_ = 9.41 μmol/L	Inhibiting PI3K/AKT/mTOR signaling pathway, blockimg G2/M phase and inducing apoptosis via mitochondrial and death receptor pathways	Zhang (2020)
Methyl protodioscin	*In vitro*	Cervical cancer Hela cells	IC_50_ = 18.49 μmol/L	Blocking G2/M phase and inducing apoptosis via endogenous and exogenous pathways	Ma et al. (2019)
	*In vitro*	Hepatocellular carcinoma HepG2 cells Cervical cancer Hela cells	IC_50_ = 20.48 μmol/L IC_50_ = 18.31 μmol/L	Inducting cell cycle arrest and apoptosis via the mitochondrial pathway and death receptor pathway	Zhang (2020)
Homoisoflavonoids	*In vitro*	Breast cancer MCF-7 cells	8-methyl-DBP: IC_50_ = 30 μmol/L 8-methoxy-DBP: IC_50_ = 90 μmol/L	Inducting phosphorylation in tumor cells blocked in the G2/M cell cycle	Rafi and Vastano (2007)
	*In vitro*	Hepatocellular carcinoma HepG2 cells and non-small cell cancer cells	IC_50=_17.99 ± 1.45 μmol/L	Inhibiting tumor cell proliferation	Xuan et al. (2025)
Homoisoflavanone-1	*In vitro*	A549 cells	6 h IC_50_ = 49.11 μg/mL 12 h IC_50_ = 37.87 μg/mL 24 h IC_50_ = 37.11 μg/mL	Regulating mitochondrial cysteine-dependent and endoplasmic reticulum stress signaling pathways, activating p38/p53 signaling pathway, induction of apoptosis	Ning et al. (2018)
PCL	*In vitro*	A375 melanoma cells	2, 6, 12, 24, 48 μg/mL for 48h	Inducting autophagy and apoptosis	Liu et al. (2009b)
	*In vitro*	LNCap cells	1, 10, 50, 100 μg/mL	Regulating caspase and Bcl-2 protein expression and inducing apoptosis	Sun et al. (2022)
	*In vitro*	L929 fibrosarcoma cells	IC_50_ = 15,000 μg/mL	Activates caspase-9, caspase-8 and caspase-3 proteins and inducing apoptosis	Zhang et al. (2010)
	*In vitro*	A375 melanoma cells	IC_50_ = 15 μg/mL	Inducting autophagy	Liu et al. (2009a)
	*In vitro*	PC3 prostate cancer bone metastasis cells	6.25, 12.5, 25, 50, 100, 200, 400, 800 μg/mL	Suppressing tumor cell energy metabolism	Zhang et al. (2017)

### 4.1 Arresting the tumor cell cycle

The cell cycle of tumor cells resembles that of normal cells, comprising the G1, S, G2, and M phases; however, its regulatory mechanisms are frequently disrupted by genetic mutations and other factors, resulting in uncontrolled proliferation (Matthews et al., 2022). Transitions from G1 to S phase and G2 to M phase involve complex molecular events that are sensitive to environmental stimuli and currently represent key targets in tumor therapy (Liu et al., 2022). PSPs, flavonoids, and steroidal saponins found in *Polygonatum* spp. have been shown to disrupt tumor cell cycle regulation, promoting cytotoxicity and inducing apoptosis.

In the H22 hepatocellular carcinoma xenograft model, flavonoids and steroidal saponins can induce G0/G1 phase arrest in tumor cells, thereby preventing DNA replication in the S phase and inhibiting cell proliferation (Duan et al., 2014). Studies have shown that PSP effectively induces G2/M phase cell cycle arrest in HeLa cells by downregulating the expression of CDK1 and Cyclin B1 (Li et al., 2020). Additionally, research indicated that dioscin, another bioactive metabolite of *Polygonati rhizoma*, arresting the G2/M phase of tumor cells through the upregulation of p53, Caspase-3, Caspase-9, and Bax, concomitant with the downregulation of Bcl-2 (Luo, 2024). Furthermore, investigations have revealed that methylprotodioscin, a major saponin metabolite, significantly restrained the proliferation of HeLa cells via multiple mechanisms, including the induction of G2/M phase arrest, the enhancement of intracellular ROS accumulation, and the activation of the death receptor pathway (Ma et al., 2019).

### 4.2 Induction of tumor cell apoptosis

In oncology, apoptosis is an essential procedure of programmed cell death that inhibits tumor progression and maintains tissue homeostasis (Nagata, 2018). Three major apoptotic pathways have been characterized: the extrinsic pathway (death receptor-mediated), the intrinsic pathway (mitochondria-mediated), and the endoplasmic reticulum stress pathway. Within these pathways, the caspase protease family and the Bcl-2 protein family serve as crucial regulators of tumor cell apoptosis (Qian et al., 2022; Sahoo et al., 2023).

Water-soluble PSP derived from *Polygonatum sibiricum* Redouté could efficiently induce apoptosis in HepG2 hepatocellular carcinoma cells, mediated by activation of the mitochondrial apoptotic pathway, characterized by stimulation of Bax, suppression of Bcl-2, and increased activities of caspase-3 and caspase-9. These molecular changes ultimately led to DNA fragmentation and nuclear damage (Li et al., 2022). Methylprotodioscin isolated from *Polygonatum sibiricum* Redouté elicited apoptosis in HeLa cervical cancer cells via dual mechanisms involving the mitochondrial pathway and the death receptor pathway (Zhang, 2020). Homoisoflavanone-1, purified via ethanol extraction, significantly reduced the proliferation of tumor cells and induced apoptosis in a dose-dependent manner in A549 cells (Ning et al., 2018). This effect was attributed to several mechanisms, including the intervention of mitochondrial cysteine-associated pathways and endoplasmic reticulum (ER) stress signaling, the activation of the p38/p53 signaling axis, and which ultimately led to the induction of apoptosis in A549 cells. Similarly, PLC has been proved to induce apoptosis in L929 murine fibrosarcoma cells, with an IC50 value of 15 μg/mL. This process was primarily mediated through the activation of caspase-9, caspase-8, and caspase-3 (Zhang et al., 2010).

### 4.3 Inhibition of tumor cell proliferation

Tumor cell proliferation refers to an abnormal and rapid process of cell division that bypasses normal growth regulatory mechanisms. It is characterized by autonomy, an accelerated cell cycle, evasion of growth inhibition, metabolic reprogramming, spatial heterogeneity, clonal evolution, and the ability to invade and metastasize, ultimately resulting in tumor formation and progression (Keibler et al., 2016). PSP has been explored for its effects on the human esophageal squamous cell carcinoma (ESCC) cell line Eca109 using *in vitro* assays at gradient concentrations of 50, 100, 200, and 400 μg/mL. This substantially attenuated the proliferative capacity of Eca109 cells by regulating TLR4 expression and inhibiting the NF-κB signaling pathway (Zhou et al., 2019b). Moreover, PCL significantly modulated the expression of apoptosis-related proteins, specifically increasing Bax levels while diminishing Bcl-xL and Bcl-2 expression. This alteration led to the excessive accumulation of ROS within the mitochondrial pathway, subsequently activating key signaling molecules such as p38 and p53. The resulting signaling cascade effectively suppressed the proliferation of human melanoma A375 cells, with an IC50 value of 15 μg/mL observed within 24 h (Liu et al., 2009a).

### 4.4 Induction of tumor cell autophagy

Autophagy is an evolutionarily conserved intracellular catabolic mechanism characterized by the formation of double-membraned autophagosomes, which engulf defective organelles and aggregated proteins and subsequently fuse with lysosomes to enable their degradation and recycling. This tightly regulated process is essential for maintaining cellular homeostasis, as it removes impaired cellular metabolites and helps to sustain energy equilibrium, especially during periods of nutrient scarcity (Debnath et al., 2023). Flavonoid polysaccharides have been shown to specifically target the proliferation of prostate cancer-associated fibroblasts (CAFs), inducing apoptosis, while exerting minimal effects on normal fibroblasts. This effect was associated with elevated levels of Beclin-1 and LC3-II, key autophagy-related proteins, which in turn enhanced autophagic activity and stimulated programmed cell death in cancer cells. A dose-dependent relationship between PSP concentration and autophagy induction was observed, with maximal autophagic activity at 1,250 μg/mL (Han et al., 2016). Moreover, dioscin has been shown to induce autophagy by modulating the PI3K/AKT/mTOR and p53 signaling pathways, through downregulation of PARP and Bcl-2, upregulation of cleaved c-PARP and Bad, and an increased LC3-II/LC3-I ratio, thereby demonstrating significant antitumor activity in endometrial cancer (Li et al., 2024).

### 4.5 Antitumor angiogenesis

Vascular endothelial growth factor (VEGF) is a crucial modulator of tumor angiogenesis, exerting its effects primarily through interactions with vascular endothelial cells. It stimulates the proliferation and migration of these cells, facilitating the development of new blood vessels. This process is crucial for providing tumors with the necessary nutrients and oxygen to support their growth and metastatic potential (Geindreau et al., 2021). Studies have shown that the polysaccharide from *Polygonatum sibiricum* Redouté can efficiently suppress the expression and activity of VEGF in a dose-dependent manner. Specifically, at a concentration of 100 μg/mL, PSP significantly reduced VEGF secretion compared to the control group. This finding suggested that PSP could effectively inhibit VEGF production by hepatocellular carcinoma cells, ultimately leading to the suppression of tumor angiogenesis (Zhang, 2025).

### 4.6 Regulation of immune function

The immune system plays a crucial role in defending against tumor development by identifying and eliminating abnormally proliferating tumor cells, while maintaining internal homeostasis. Therefore, enhancing immune function is considered a key strategy in cancer treatment (Petroni et al., 2021). *Polygonatum* spp. Exhibits remarkable immunomodulatory properties, enhancing the functionality of immune cells such as NK cells and macrophages, thereby bolstering the body’s antitumor immune responses.

Crude polysaccharides from *Polygonatum cyrtonema* Hua have been shown to prolong the survival of mice bearing S180 ascitic tumors by enhancing immune function, with dosages of 200 mg/kg and 400 mg/kg exceeding the efficacy thresholds defined in traditional Chinese medicine (Ye et al., 2008). Further studies demonstrated that PSP could inhibit the proliferation of hematopoietic cells in the spleen induced by triple-negative breast cancer (TNBC), and significantly increase the number of hematopoietic stem and progenitor cells (HSPCs) as well as common lymphocytes in the bone marrow (Xie et al., 2021). PSP also significantly restrained tumor expansion in a murine model of gastric cancer, likely via the suppression of the TLR4/NF-κB signaling pathway. This suppression enhanced the immunoregulatory balance of cytokines, including TNF-α, IL-2, and IL-6, thereby impeding tumor progression (Lv and Duan, 2020). Additionally, PSP stimulated the proliferation and differentiation of immune cells by activating the TLR4 receptor and its downstream MAPK/NF-κB signaling pathway, augmenting immune responsiveness and enhancing tumor cell recognition and cytotoxic activity (Long et al., 2018). PSP was further shown to efficiently prevent the depletion of HSPCs and lymphoid progenitor cells induced by TNBC, while modulating immunosuppressive conditions within the tumor microenvironment (Xie et al., 2021). It also regulated the spatial distribution of immunosuppressive myeloid cells in the tumor microenvironment and exert protective effects by maintaining splenic immune cell homeostasis. In addition, steroidal saponin can exert antitumor effects by modulating immune responses and enhancing host resistance to tumor development (Zhai, 2024).

### 4.7 Other antitumor mechanisms

In addition to the above pathways, the antitumor active metabolitess of *Polygonatum* spp. can also influence tumor cell energy metabolism through modulation of associated signaling cascades. In the context of prostate cancer treatment, through multiple synergistic mechanisms, PSP effectively suppressed the activation of the PI3K/AKT and NF-κB signaling pathways, downregulating the phosphorylation of PI3K, AKT, and p65, thereby promoting tumor cell apoptosis and inhibiting growth. Concurrently, Caspase-3 expression was upregulated, and the concentrations of immunomodulatory cytokines in the blood—such as TNF-α, IL-2, and IL-6—were regulated synergistically (Yang et al., 2025). *Polygonatum* spp. was found to modulate tumor progression and interfere with tumor cell energy metabolism (Yang et al., 2025). Furthermore, PCL also exerted anti-metastatic effects by inhibiting tumor cell aggregation and suppressing glycolysis in PC3 prostate cancer cells with bone metastasis, primarily through downregulation of hexokinase 2 (HK2), a key enzyme in the Warburg effect (Zhang et al., 2017). In addition, the Ras–Raf and PI3K–AKT signaling pathways also serve as critical negative regulators in the anti-tumor mechanism of *Polygonatum* spp. (Liu et al., 2010).

## 5 Combination medication

Multi-drug combination strategy is a crucial approach for improving cancer treatment, and it represents a mainstream modality in clinical oncology. TCM is characterized by its moderate efficacy, holistic treatment philosophy, and relatively low toxicity, which addresses the limitations of conventional therapies and is increasingly recognized as a valuable adjunct in tumor management. Recent studies have indicated that active metabolites of *Polygonatum* spp. can significantly enhance therapeutic outcomes when used in combination with conventional chemotherapeutic agents. For instance, co-administration of PSP with cyclophosphamide has been shown to reduce toxicity and reverse cisplatin resistance, whereas combination with Astragalus polysaccharides resulted in a synergistic enhancement of antitumor activity (Table 2).

**TABLE 2:** Anti-tumor effects of chemical metabolites of *Polygonatum* spp. in combination with drugs.

Drug combination	Study type	Cancer model	Pathway targeted	Reference
PSP- cisplatinum	*In vivo*	H22 liver cancer xenograft mice	PSP enhanced the immune system function and antioxidant capacity, synergistically inhibited the division of tumor cells and induced apoptosis of tumor cells	Li et al. (2016)
PSP- cyclophosphamide	*In vitro* an *in vivo*	Hepatocellular carcinoma H22 cells and macrophage RAW264.7 H22 liver cancer xenograft mice	Inhibiting the proliferation of liver cancer cells and antagonize the inhibition of cyclophosphamide on macrophages Enhancing the antitumor effect of cyclophosphamide, improving immune and organ function, and regulating inflammatory factors	Peng (2024)
Steroidal saponin - cyclophosphamide	*In vivo*	Cyclophosphamide-induced immunosuppressive mouse model	Enhancing immune cell proliferation, regulating inflammatory factors, repairing intestinal mucosa proteins, and boosting antioxidant effects	Zhao et al. (2024)
*Polygonatum rhizoma*-*Astragalus* *mongholicus* *Bunge*	*In vitro*	Lung cancer h1299 cells	Regulating PI3K/AKT pathway induces mitochondrial pathway apoptosis in lung cancer cells	Liu et al. (2023)
*Polygonatum rhizoma*-*Astragalus* *mongholicus* *Bunge*	*In vitro* an *in vivo*	A549 and LLC lung cancer cells; lung cancer mouse model	Inducing ferroptosis in lung cancer cells	Zhang et al. (2023)
*Polygonatum rhizoma*-*Astragalus* *mongholicus* *Bunge*	*In vivo*	Lung carcinoma *in situ* mice	Down-regulating apelin-PGC1α-UCP1 signaling pathway	Wang (2024)
*Polygonatum* Rhizoma *rhizoma*, *Morinda officinalis* (F.C.How) Razafim &B.Breme*r*, *Angelica dahuricae* (Hoffm.) Benth. & Hook.f. Ex Franch. & Sav.	*In vitro*	Eca-109 cells, HGC-27 cells, HCT-8 cells	Blocking the cell cycle in S phase	Wan et al. (2024)

### 5.1 Combinations with chemotherapeutic agents

Cyclophosphamide and cisplatin are widely utilized as chemotherapeutic agents for treating a variety of cancers. Nevertheless, their clinical use is frequently restricted due to the significant adverse effects, including immunosuppression, gastrointestinal injury, and hematological abnormalities, which collectively impair immune function and increase susceptibility to infection (Spears et al., 2019). Studies have shown that the co-administration of *Polygonatum* spp. with these agents not only enhances the chemosensitivity and mitigates toxicity to healthy tissues, but also reinforces the inhibitory influences on tumor cells. The combination of PSP with low-dose cisplatin effectively suppressed the growth of H22 hepatocellular carcinoma xenografts in mice, with the synergistic anti-tumor effect attributed to the reduction of oxidative stress (Li et al., 2016). Furthermore, PSP combined with cyclophosphamide enhanced the inhibitory effect on H22 solid tumors while simultaneously reducing the toxicity typically associated with chemotherapy (Peng, 2024). Steroidal saponins were found to alleviate cyclophosphamide-induced immunosuppression and enhance its antitumor activity through multiple mechanisms, including improvement of immune organ indices, stimulation of lymphocyte proliferation and differentiation, regulation of inflammatory cytokines, and alleviation of oxidative stress (Zhao et al., 2024).

### 5.2 Co-administration with other botanical drugs

Considering the complexity of cancer pathogenesis, monotherapy often falls short in achieving optimal therapeutic outcomes. The theory of TCM combination therapy emphasizes t synergistic effect between the botanical metabolites. This approach can effectively improve the efficacy, reduce the toxicity, and broaden the therapeutic window through multi-metabolite, multi-target, and multi-pathway mechanisms (Zhou et al., 2017). As a multi-metabolite, multi-target traditional Chinese medicine, *Polygonatum* spp. is well-suited for such strategies. Its co-administration with other Chinese botanical drugs can produce significant synergistic effects, offering novel strategies and methods for cancer treatment.

Recent pharmacological investigations have further elucidated the therapeutic potential of *Polygonatum* spp. when incorporated into combination therapies, especially in the intervention of lung cancer. Steroidal saponins were found to alleviate cyclophosphamide-induced immunosuppression and enhance its antitumor activity through multiple mechanisms, including improvement of immune organ indices, stimulation of lymphocyte proliferation and differentiation, regulation of inflammatory cytokines, and alleviation of oxidative stress (Liu et al., 2023; Zhang et al., 2023). The *Polygonatum sibiricum* Redouté–*Astragalus mongholicus* Bunge [*Fabaceae*; *Astragalus*] metabolites have been shown to inhibit mitochondrial uncoupling, restore oxidative phosphorylation, suppress aerobic glycolysis, and reverse the Warburg effect by downregulating the apelin–PGC1α–UCP1 signaling pathway, effectively suppressing lung cancer progression (Wang, 2024).

In recent years, advances in network pharmacology and molecular docking techniques have significantly advanced research into the combination of flavonoids with other traditional Chinese medicines in the field of cancer therapy. The *Polygonati rhizoma*–*Lilium brownii* var. *viridulum* Baker [*Liliaceae; Lilium*] pair was predicted to exert anticancer effects by arresting the tumor cell cycle, inducing apoptosis and autophagy, enhancing immune function, and modulating relevant signaling pathways (Yu et al., 2020). The *Panax ginseng* C. A. Mey [*Araliaceae; Panax*]–*Polygonati rhizoma* pair was shown to alleviate cancer-related fatigue by modulating multiple targets and pathways associated with apoptosis, metastasis, and inflammation in cancer cells (Jiang et al., 2022). Various concentrations of *Polygonatum rhizoma*, *Morinda officinalis*, and *Angelicae dahuricae* (Hoffm.) Benth. & Hook.f. Ex Franch. & Sav. [*Apiaceae; Angelicae dahuricae Radix*], whether used in pairwise combinations or as a formulation of three botanical drugs, effectively induced S-phase arrest and promoted apoptosis in tumor cells (Sun, 2012). Additionally, the combination of *Polygonatum sibiricum* Redouté and *Polygonatum odoratum* (Mill.) Druce has been suggested to enhance immune responses, offering new therapeutic insights for cancer treatment (Zhuang and Wang, 2019).

## 6 Discussion

Despite continuous breakthroughs in cancer therapy, a definitive solution for completely eradicating cancer has yet to be achieved. Chemotherapy remains one of the primary treatment modalities, making substantial contributions to tumor control. However, its inherent limitations—such as poor targeting and pronounced toxicity—have significantly hindered its broader clinical application. In this context, Traditional Chinese Medicine (TCM) has emerged as a promising field in oncology, owing to its multi-metabolite nature, multi-target mechanisms of action, immunomodulatory effects, and relatively low toxicity.

Among TCM botanical drugs, *Polygonatum* spp. has attracted increasing attention in recent years due to its significant progress in antitumor research. Extensive phytochemical investigations have isolated a diverse range of bioactive metabolites in *Polygonatum*, including polysaccharides (PSP), steroidal saponins, flavonoids, volatile metabolites, and PCL, all of which exhibit strong antitumor activity. These metabolites exert synergistic effects through multiple mechanisms, including cell cycle arrest, apoptosis promotion, autophagy induction, tumor proliferation suppression, and immune modulation, demonstrating substantial therapeutic potential.

However, despite these promising findings, *Polygonatum* spp. research faces several critical limitations that warrant further investigation.

Firstly, although the antitumor properties of key metabolites such as PSP, steroidal saponins, and flavonoids have been established, most studies to date have focused on isolated targets or signaling pathways, lacking a comprehensive understanding of their multi-target interactions. Future studies should adopt integrative approaches, including network pharmacology and omics technologies, to elucidate the complex molecular mechanisms underlying the actions of *Polygonatum* spp., especially its flavonoid metabolites.

Secondly, there is an urgent need to strengthen *in vivo* and clinical investigations. Most existing studies have been limited to *in vitro* experiments, and *in vivo* efficacy and clinical outcomes remain underexplored. Animal studies and rigorously designed clinical trials are essential to confirm the safety and efficacy of *Polygonatum* spp., thereby facilitating its transition into clinical application.

Thirdly, the quality control system for *Polygonatum* spp. requires substantial refinement. The l metabolite of *Polygonatum* spp. is influenced by multiple factors, including geographical origin, cultivation conditions, harvesting season, and processing methods. These variables can affect the content, stability, and bioactivity of its metabolites, thereby influencing its pharmacological properties. It is imperative to establish standardized cultivation techniques, processing protocols, and quality control methods to ensure the reproducibility and consistency of its bioactive metabolites.

Lastly, more research should be dedicated to optimizing drug combination strategies. While current studies have demonstrated promising synergistic effects of *Polygonatum* spp. in combination with chemotherapeutic agents, pharmacokinetic and pharmacodynamic data remain scarce. Future research should address these aspects to ensure the safety and efficacy of combination therapies.

With the increasing global recognition of TCM, *Polygonatum* spp. holds significant promise, not only as a valuable therapeutic agent but also as part of integrative oncology treatments. Its application could bridge the gap between traditional and modern cancer therapies, making it a promising candidate in the global fight against cancer. Priority areas for future research include: (1) clarifying multi-target interactions via systems biology and omics-based approaches; (2) expanding *in vivo* and clinical studies with a focus on pharmacokinetics, pharmacodynamics, and safety; (3) establishing standardized cultivation, processing, and quality control methods; and (4) developing mechanism-based and clinically validated combination strategies with chemotherapeutics and other TCM botanical drugs.

In summary, *Polygonatum* spp., as a medicinal and edible plant with significant antitumor potential, represents a valuable candidate for future cancer therapy. However, its successful clinical application will depend on continued exploration in key areas, including mechanistic elucidation, clinical validation, standardization, and combination therapy optimization. Through sustained and rigorous scientific inquiry, the full therapeutic potential of *Polygonatum* spp. can be realized, ultimately offering cancer patients safer, more effective, and integrative treatment options.
